# A correlation analysis of Light Microscopy and X-ray MicroCT imaging methods applied to archaeological plant remains’ morphological attributes visualization

**DOI:** 10.1038/s41598-020-71726-z

**Published:** 2020-09-15

**Authors:** Cristina Marilin Calo, Marcia A. Rizzutto, Sandra M. Carmello-Guerreiro, Carlos S. B. Dias, Jennifer Watling, Myrtle P. Shock, Carlos A. Zimpel, Laura P. Furquim, Francisco Pugliese, Eduardo G. Neves

**Affiliations:** 1 Laboratory of Archaeometry and Applied Sciences To Cultural Heritage (LACAPC), Institute of Physics, University of Sao Paulo, Sao Paulo, Brazil; 2 Department of Plant Biology, Institute of Biology, University of Campinas (UNICAMP), Campinas, Brazil; 3 Brazilian Synchrotron Light National Laboratory (LNLS), Brazilian Centre for Research in Energy and Materials (CNPEM), Campinas, Brazil; 4 Museum of Archaeology and Ethnology (MAE), University of Sao Paulo, Sao Paulo, Brazil; 5 Federal University of Western Para (UFOPA), Santarem, Brazil; 6 Department of Archaeology, Federal University of Rondonia Foundation (UNIR), Porto Velho, Brazil; 7 Institute of Geosciences, University of Brasilia, Brasília, Brazil

**Keywords:** Biological techniques, Imaging, Microscopy, Natural variation in plants

## Abstract

In this work, several attributes of the internal morphology of drupaceous fruits found in the archaeological site Monte Castelo (Rondonia, Brazil) are analyzed by means of two different imaging methods. The aim is to explore similarities and differences in the visualization and analytical properties of the images obtained via High Resolution Light Microscopy and X-ray micro-computed tomography (X-ray MicroCT) methods. Both provide data about the three-layered pericarp (exo-, meso- and endocarp) of the studied exemplars, defined by cell differentiation, vascularisation, cellular contents, presence of sclerenchyma cells and secretory cavities. However, it is possible to identify a series of differences between the information that can be obtained through each of the methods. These variations are related to the definition of contours and fine details of some characteristics, their spatial distribution, size attributes, optical properties and material preservation. The results obtained from both imaging methods are complementary, contributing to a more exhaustive morphological study of the plant remains. X-ray MicroCT in phase-contrast mode represents a suitable non-destructive analytic technique when sample preservation is required.

## Introduction

The identification of the taxonomic provenance and preservation conditions of plant remains recovered from archaeological deposits is at the basis of archaeobotanical research. The whole procedure involves a detailed description of observable characteristics of the morphological phenotype of the sample, together with a comparative analysis of modern and ancient exemplars of related taxa.

On the other hand, plant remains from archaeological sites are not always recognizable through their external morphological characteristics, for reasons that include the presence of similar traits between different species, morphological changes induced by taphonomic processes, and other issues which hinder the identification of specimens. In these cases, the analysis of the internal characteristics of the specimen can increase the amount of morphological data used to determine the anatomic and taxonomic provenance of ancient plant remains.

In general, Archaeobotany has a wide palette of microscopy techniques that allow for various descriptions of both external and internal morphological features of archaeological plant samples^1,2^. In fact, it is possible to obtain very detailed high resolution images of studied objects using some optical or electronic microscopy methods. Nevertheless, these images are limited to regions near the surface of the sample and/or to thin transparent or semitransparent samples. Internal structure and organization of thick and opaque objects are accessible only using a combination of microscopy, staining and microtome techniques, all of these having a necessary impact on sample integrity^3,4^.

By contrast, no destructive sample preparation procedure is required for high resolution X-ray MicroCT. Volume reconstruction and virtual slicing prevent manipulation damage and the loss of material and information. As with staining, digital segmentation allows distinguishing and analyzing any feature separately, based on the image contrast.

As a whole, the use of highly detailed 3D X-ray MicroCT images to study the internal structure of materials and objects is a growing and promising area in scientific research. Non-destructiveness is one of the most remarkable advantages of this method and its relevance increases when samples are rare, non-reproducible or very fragile. For this reason, X-ray MicroCT has been suggested as a suitable and useful procedure for applications on historical, artistic and culturally valuable objects made from diverse materials^5^. Several ethnographic and historical items manufactured on raw plant materials have been studied by X-Ray MicroCT i.e.^6–8^. Archaeological application of this technique on plant remains includes the analysis of contaminants in charred remains dated by radiocarbon^9^, dendrochronology^10^, wood characterization i.e.^11,12^ and identification of carpological remains^13,14^.

However, so far, no studies have been conducted to assess the extent to which the X-ray MicroCT method can provide, by itself, the appropriate data for addressing issues of plant remains identification. In other words, if it is possible to obtain morphological information that is quantitatively and qualitatively comparable to that accessible by more regularly used light microscopy methods This study seeks to shed light on how three-dimensional data on the internal structure of plant remains contribute to the morphological examination of plant remains and what challenges arise from their application in Archaeobotany.

This article is based on a previous X-ray MicroCT analysis of non-charred plant remains from the archaeological site Monte Castelo (Rondonia, Brazil). The results of that study were especially relevant to question the hypothesis of the caryopsis (maize-type) morphology, externally observed in several exemplars. In contrast, the technique allowed the identification of their drupaceous anatomy, and the suggestion of some morphological relationships with the Anacardiaceae^15^. Those same specimens were imaged using X-ray MicroCT and High Resolution Light Microscopy (HRLM) to specify the presence of a three-layered pericarp, vascular bundles and differentiated cellular contents. This essentially methodological contribution is aimed to explore variations in the visualization and analytical scope of both techniques, focusing on these morphological attributes.

## Methodology and materials

### The samples

**Figure 1:**
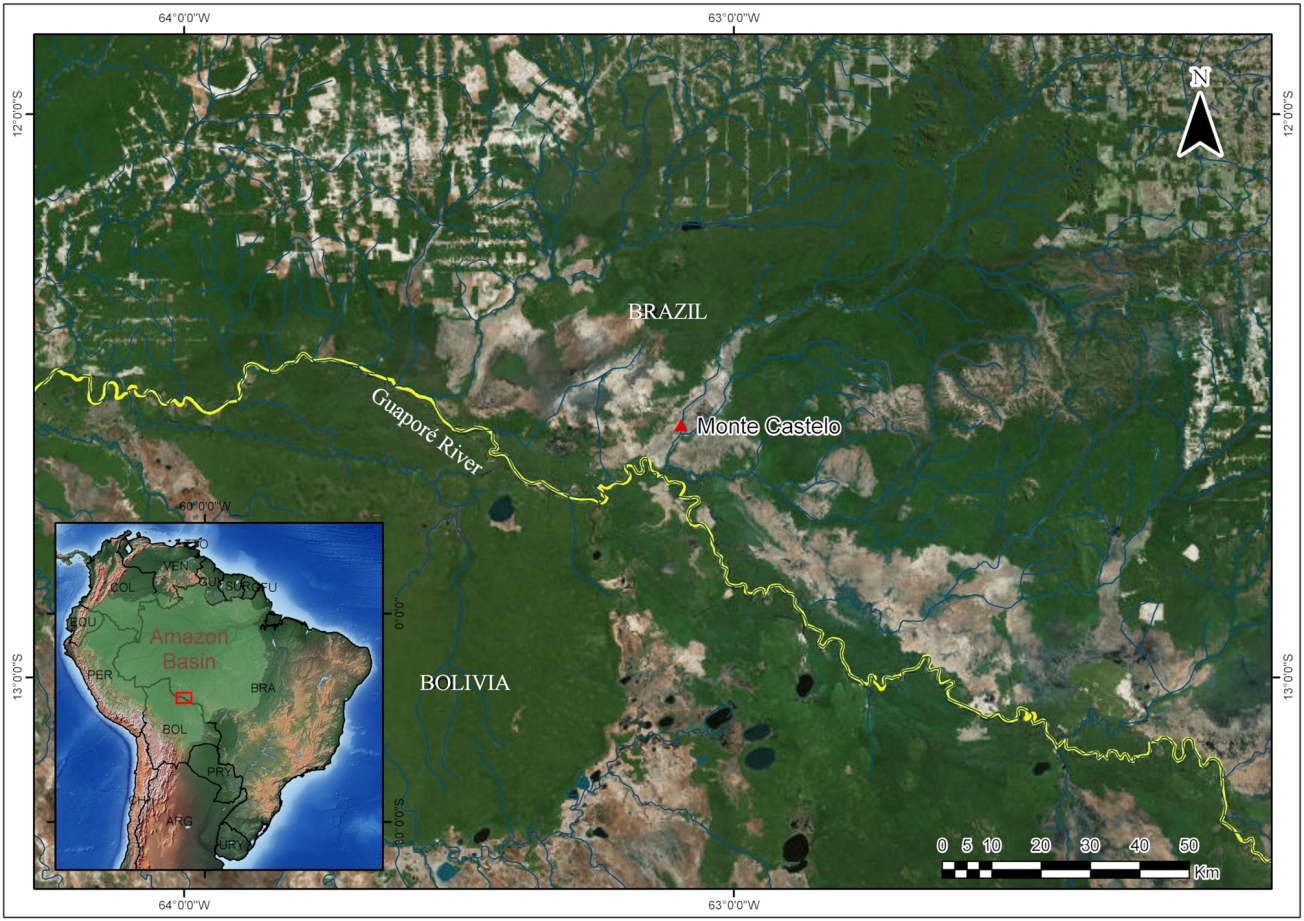
The location of the Monte Castelo archaeological site in the Middle Guapore River basin area (adapted)^18^ (QGis version 3.8, URL: https://www.qgis.org).

This work compares the results obtained from the application of two alternative imaging techniques, HRLM and X-ray MicroCT, to the analysis of internal morphological characteristics of three plant remains samples. The specimens SMC_01, SMC_10 and SMC_11 were recovered at the archaeological site Monte Castelo, located in the floodplain of the Middle Guapore River, in the southwestern Brazilian Amazon. The site is an artificial mound with dimensions of ca. 120 m by 100 m and a height of 6.3 m, constructed in an area of ecological transition between periodically flooded savannahs and high-ground, evergreen tropical forest. Archaeological excavations reveal an uninterrupted sequence of occupation events showing evidence of several domestic activities dating from ca. 6,000 years BP^16–19^ (Fig. 1).

**Figure 2:**
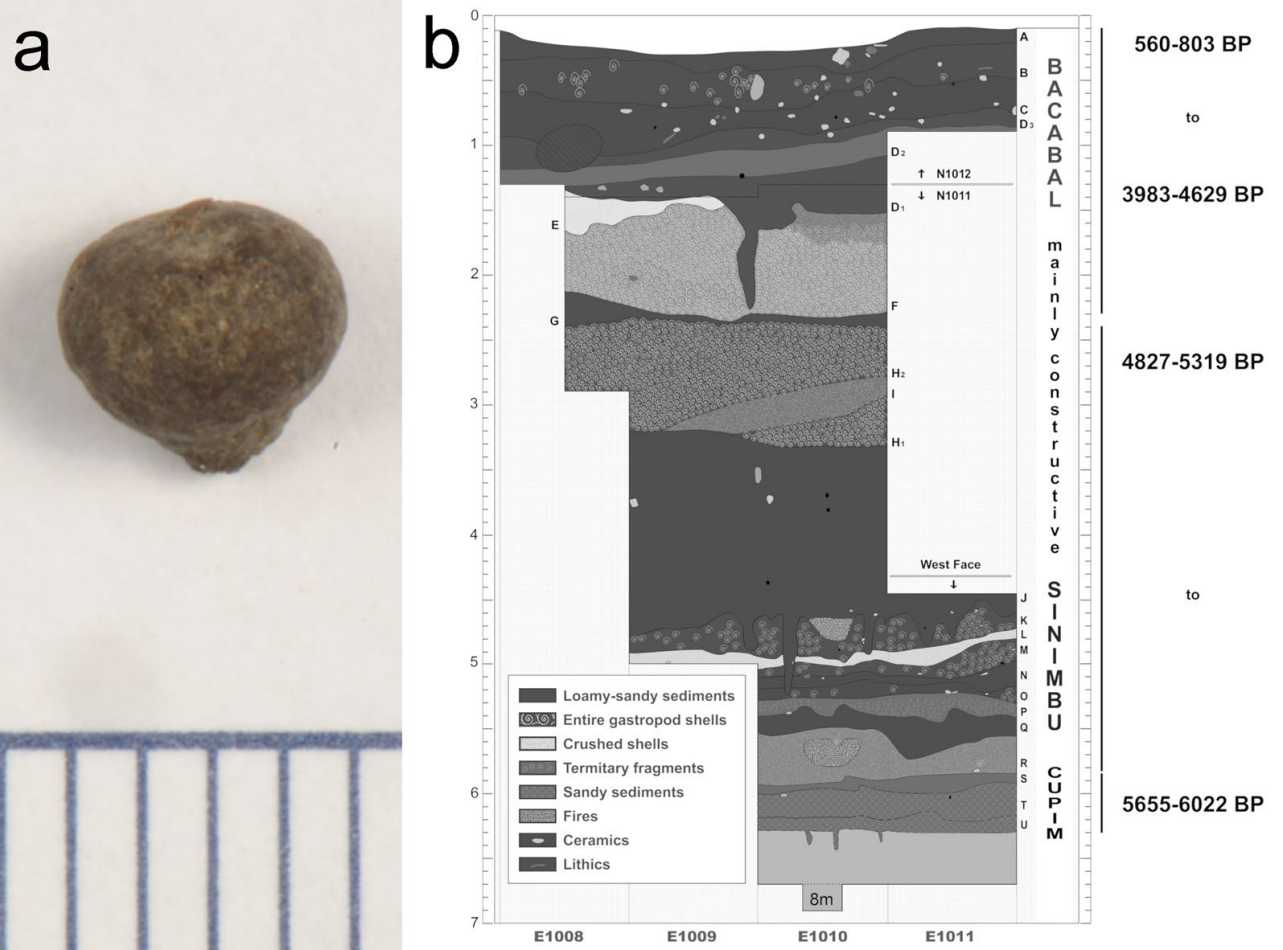
(**a**) Photomicrography of the sample SMC_10 (scale in mm) (Photo: Pedro Campos—Laboratory of Archaeometry and Applied Sciences to Cultural Heritage, Institute of Physics, University of Sao Paulo); (**b**) scheme of the north profile of the Monte Castelo shellmound and radiocarbon dates (cal. 2-sigma) (adapted)^18^.

The samples were selected from a large set of dried, non-charred, small drupe fruits (Fig. 2a). This botanical material comes from soil collections made after the definition of the cultural layers and, therefore, have secure provenience. These fruits were found concentrated in the most recent stratigraphic package of the shellmound that is related to the Bacabal Archaeological Phase, although they are also present in other archeological layers. This Phase consists predominately of layers of dark soil and shells, where richly decorated pottery, animal bones, plant materials, and human burials have been found^15,18–21^ (Fig. 2b).

Examined with the naked eye and low magnification optics, the samples have globular to sub-globular shapes with pointy ends and rather irregular surfaces. The average maximum length and diameter of the studied exemplars are 4.9 mm and 4.6 mm respectively, while volume ranges between 33.3 to 40.4 mm^3^. Longitudinal lines or furrows are present, extending from the pointy end to approximately the medial region. Opposite to the pointy end, the rounded contour is only interrupted by a marked protuberance.

### Imaging methods

The methodology is focused on the application of two different imaging methods X-ray Computed Microtomography (X-ray MicroCT) and High Resolution Light Microscopy (HRLM), whose performance for visualization and analysis are compared on the basis of a specific set of drupe morphological characteristics (see “Referenced internal morphological attributes of drupes” section).

For the first imaging method, tomographic 3D images at a micrometer scale of samples SMC_01 and SMC_10 were reconstructed based on the X-ray MicroCT technique in propagated-based phase-contrast mode at the IMX Beamline of the Brazilian Synchrotron Light Laboratory (LNLS-CNPEM). The technical features of the IMX Beamline include the detector PCO pco.2000 area camera, 0.82 μm effective pixel size, 2048 × 2048 pixel, 14-bit CCD cooled camera.

For both experiments an X-ray energy range (pink-beam) 4 to 14 keV was selected. The exposure time/projection varied from 0.4 s in SMC_01 to 0.5 s in SMC_10. The sample-to-detector distance and the angular range were established at 260 mm and 360°, respectively. The effective pixel size was 0.82 μm for a total of 2048 projections on each microtomographied sample. A 350 μm Si filter was used in both cases. These parameters were set based on a previous test imaging experiment, performed on the same exemplar SMC_01 at the IMX Beamline, and another 10 specimens imaged using industrial microtomographers (Calo et al. 2019). They were selected in order to optimize resolution and contrast for the minimum necessary scanned area of the sample to reveal internal specific characteristics and the global time requirements to complete the experiment (delimited by the synchrotron experiments dynamic).

X-ray MicroCT images result from the application of mathematical algorithms^22^ to X-ray experimental data, obtaining a sequence of 2D images (slices) and stacked together to reconstruct a 3D map of the object’s X-ray attenuation coefficient (transmission or absorption tomography). If certain experimental conditions are fulfilled, then phase shifts of the transmitted X-rays can also be detected. Absorption and phase modulation are effects that occur on X-rays crossing any kind of materials. The use of phase sensitive imaging techniques has the advantage of enhancing the visibility of low absorption materials as well as accentuating the edges between materials with small differences in refraction index. This requires a highly spatially-coherent X-ray beam provided mostly by synchrotron light sources^5,23,24^, but also by microfocus X-ray sources^25^. This study used the X-ray propagation based—phase contrast MicroCT via synchrotron radiation^26,27^.

The in-house software Raft, developed at LNLS-CNPEM^28–30^, was applied to reconstruct data acquired from the IMX Beamline. The Paganin filter (sometimes considered a PCI projection restoration)^31^, regularly implemented in the IMX Beamline reconstruction software for edge enhancement of low contrast samples, were applied to projections acquired in this study. Visualization, segmentation and 3D morphological analysis of microtomographic images were achieved using CT Analyzer 1.15.4.0, developed by Bruker^32^ and the FIJI distribution version 1.52p of the image processing software ImageJ^33–35^ with the plug-ins MorphoLibJ^36^, 3DViewer^37^ and Volume Viewer^38^. All figures were edited using GNU Image Manipulation Program (GIMP) in the version 2.10.2 (https://www.gimp.org/).

For the HRLM imaging method, 2D photomicrography of the internal morphological characteristics of the sample SMC_11 were examined and captured using two different microscopes. The first of them was an Olympus System Microscope model BX51 coupled to an Olympus Microscope Digital Camera Model DP71 in the Plant Anatomy Laboratory of the Biology Institute, University of Campinas. The second was an Erns Leitz GmbH microscope model Ortoplan-Pol coupled to a Kodak Digital Camera model DC4800 in the Laboratory of Complex Fluids of the Institute of Physics, University of Sao Paulo.

As non-destructiveness (including damage or modification) is considered a relevant issue of the imaging method for plant remains, only one specimen was subjected to the HRLM method of sample preparation in the Plant Anatomy Laboratory of the Biology Institute, University of Campinas. The specimen SMC_11 was previously fixed in FAA (formaldehyde, acetic acid, 50% ethanol) for 24 h^39^. Then, it was dehydrated in an ethanol series and embedded in hydroxyethyl methacrylate resin (Historesin Leica)^40^. A total of 96 longitudinal slices of 8.0 µm thick were obtained from one of the halves of the lengthwise divided sample, using a Microm HM340E rotary microtome. The slices were stained with 0.05% Toluidine Blue in sodium acetate buffer pH 4.7^41^ and fixed to microscope slides. All slices were mounted with water for examination.

## Referenced internal morphological attributes of drupes

The internal characteristics considered here for the analysis of the fruit remains comprises the three-layered pericarp (endocarp, mesocarp and exocarp) defined by cell differentiation, vascularisation, presence of cellular contents, sclerenchyma cells, cavities and ducts. This set of descriptive variables is based on specific studies on fruit anatomy and classification^42–44^. In general, drupes can be distinguished from other fruits by their fleshy mesocarp and hard endocarp tissues. The peach could be considered a typical example of a drupe^42,43^.

The exocarp (*strictu *sensu) is usually uniseriate and, in many fruits, represents the main protective layer of the pericarp. Notwithstanding, multiseriate exocarps are very seldom found in several fruits. This layer of tissue may be composed of regular epidermal cells of isodiametric or elongated shapes in surface view, but palisade cells could appear in some drupes. The outer tangential walls of the epidermal cells are generally thick and covered by a well-developed cuticle. Stomata, lenticels and trichomes are frequently present. Anthocyanins are regularly reported as contents in the cell sap of epidermal cells, producing the red, purple, blue and black colors of the fruit. Calcium oxalate crystals, raphides, siliceous impregnations, cutines, suberines and waxes also occur in specialized epidermis cells^42,45^.

The endocarp (*strictu *sensu) is often composed of smaller cells than the exocarp, especially in fleshy fruits, and they are frequently elongated. Drupes develop a distinctive hard multilayered endocarp formed by sclerenchymatic tissue. Differently from parenchymatous endocarps, its main function is to serve as supporting tissue and to protect the seed from injuries. The sclerenchyma is formed by thick secondary walls and strongly lignified cells denominated sclereids (short cells), fibers (long cells) and fiber-sclereids. Several types of sclereids could be present in the endocarp structure. The most common are brachysclereids, or stone cells, roughly isodiametric or somewhat elongated cells; macrosclereids, represented by elongated columnar cells; and osteosclereids or bone-cells, also columnar but with enlarged ends^42,45^.

As crystals are often found in the vicinity of sclerenchymatic cells, a crystal layer frequently accompanies the endocarp in drupes^42^. Inorganic deposits on plants consist mostly of calcium salts and anhydrides of silica which occurs in many plant families. Although calcium carbonate crystals are rarely present, the crystalline form of calcium oxalate is very common. They appear as prismatic, raphide, druse and styloid crystal morphologies. In some tissues, calcium oxalate crystals arise in cells that resemble adjacent, crystal-free cells. In others, the crystals are formed in idioblasts, specialized secretory cells with a distinctive form (vesicles, sacs, or tubes). On the other hand, silica compounds are more present in cell walls or form silica bodies or phytoliths in the lumen of cells^45^.

Besides crystals, idioblasts can also produce oils, mucilage and tannins^42^ and can be distributed all over the pericarp. Other secretory structures in drupes are the cavities and ducts, formed by several specialized cells which release those secondary metabolites to the intercellular space. This characteristic is very conspicuous in the mesocarp of drupes of the Anacardiceae family i.e.^46–52^, also referred to as lacunar mesocarp^44^.

The parenchyma plays an essential part in the formation of the fleshy mesocarp of drupes. It generally consists of large rounded or elongated (in a radial direction, generally the inner mesocarp) cells with thin walls and large vacuole. In ripe drupes, these are usually distinguished by their juiciness. The cell sap of parenchyma may be enriched with anthocyanin and sugar, together with tannins, acids and other contents. Crystals of different shapes may also occur and appear in specially preformed layers. Small intercellular spaces may eventually develop between the cells. As the parenchyma represents the edible part of drupes, it functions as a storage tissue in maturing fruits; it may contain starch, sugar, oil, fats, etheric oil (deposited in idioblasts, special glands or oil ducts) and latex in laticifers. Collenchymatic tissue is also found frequently in the fleshy mesocarp of drupes. This small-celled and thick walled cells tissue occupies the periphery of the pericarp or the vicinity of vascular bundles. Its primary function is mechanical support^42,43^.

Vascular bundles are the main transport channels of water and nutrients in the fruit. They form an anastomosed network in the drupe's pericarp running through the mesocarp. The vascular supply of fruit varies according to the type of ovary from which it originates. The fleshy fruits develop an additional bundle system and the whole finer bundles are more numerous in fleshy tissues than in dry and/or stony parts^45^.

## Results

### Pericarp

**Figure 3:**
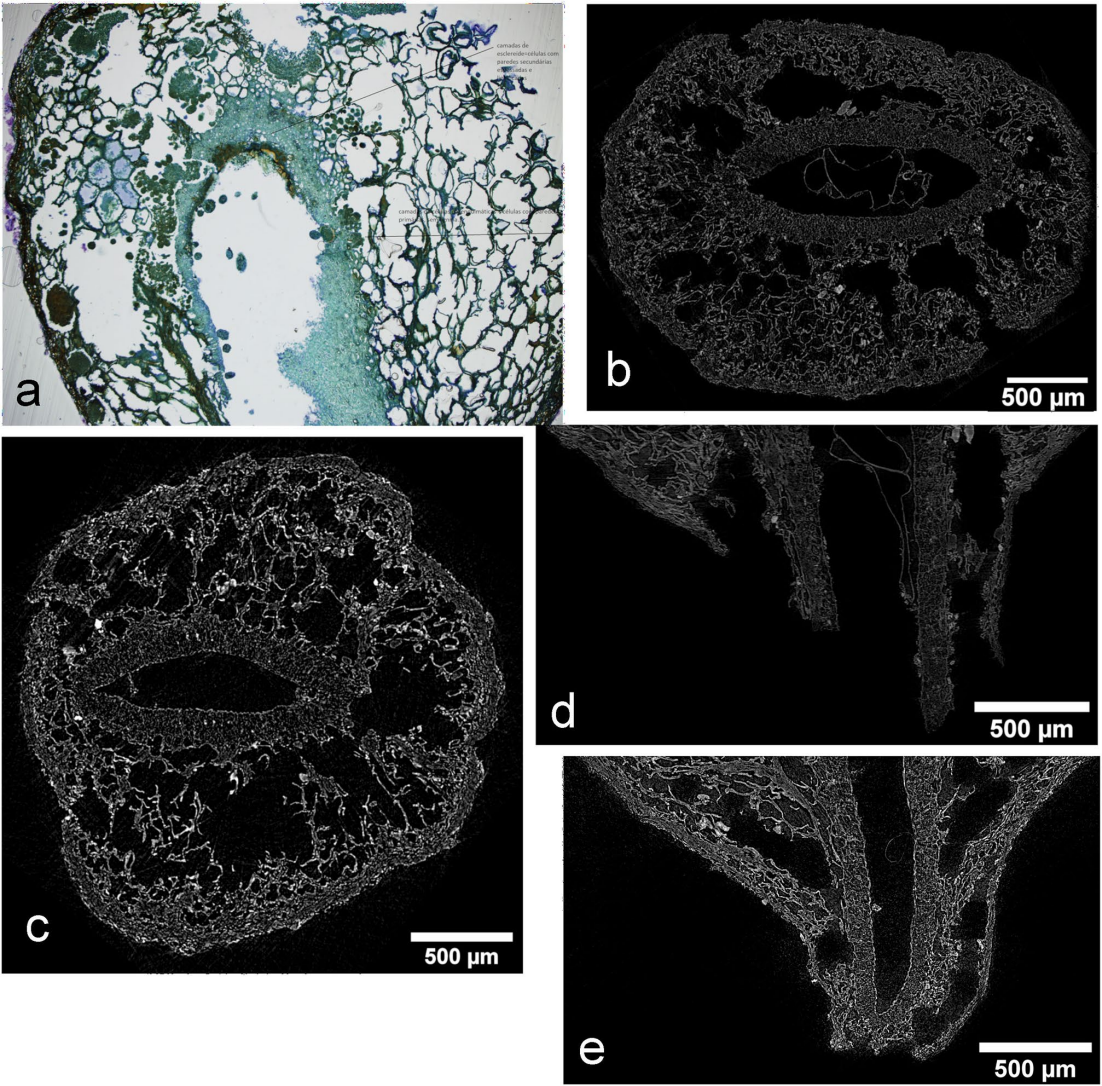
General views of the three-layered structure of the fruit as seen by HRLM image of a longitudinal microtome section from SMC_11 (**a**); virtual X-ray MicroCT transversal section from SMC_01 (**b**) and SMC_10 (**c**) idem for longitudinal section (**d**, **e**) (ImageJ-FIJI).

**Figure 4:**
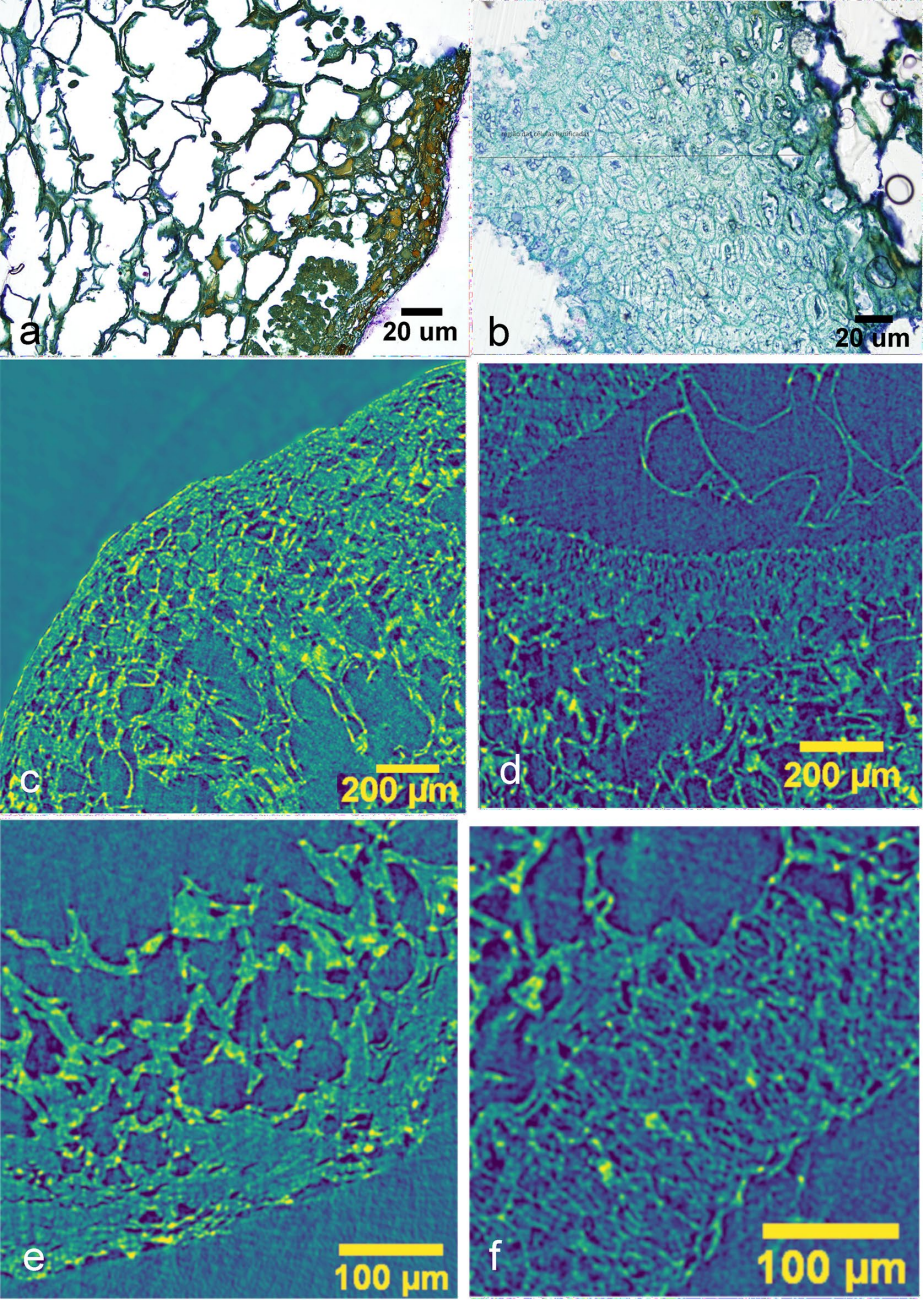
Detail of cell morphologies of the exocarp and endocarp layers: (**a**, **b**) HRLM from sample SMC_11; X-ray MicroCT transversal section; (**c**, **d**) from sample SMC_01; and (**e**, **f**) from sample SMC_10 (ImageJ-FIJI).

Both HRLM and X-ray MicroCT longitudinal sections show a three-layered internal structure marked by straight differences between cellular components (Fig. 3). In HLRM images, the exocarp corresponds to a multiseriate layer stained in brown-green. Cells are small and slightly regular with prevailing isodiametric shapes and some contents. No palisade cells were observed. A fine cuticle layer is present on the outer cells in some regions of the exocarp. The mesocarp appears as the most voluminous tissue, formed by large, irregular parenchymatic cells. Primary cell walls are well preserved (Fig. 4a). The endocarp is a multiseriate layer formed by mostly regular, small and highly lignified sclerenchymatic cells (brachysclereids) stained in green–blue (Fig. 4b). No structure related to seed-embryo tissues was observed in HRLM images from the studied exemplar.

**Figure 5:**
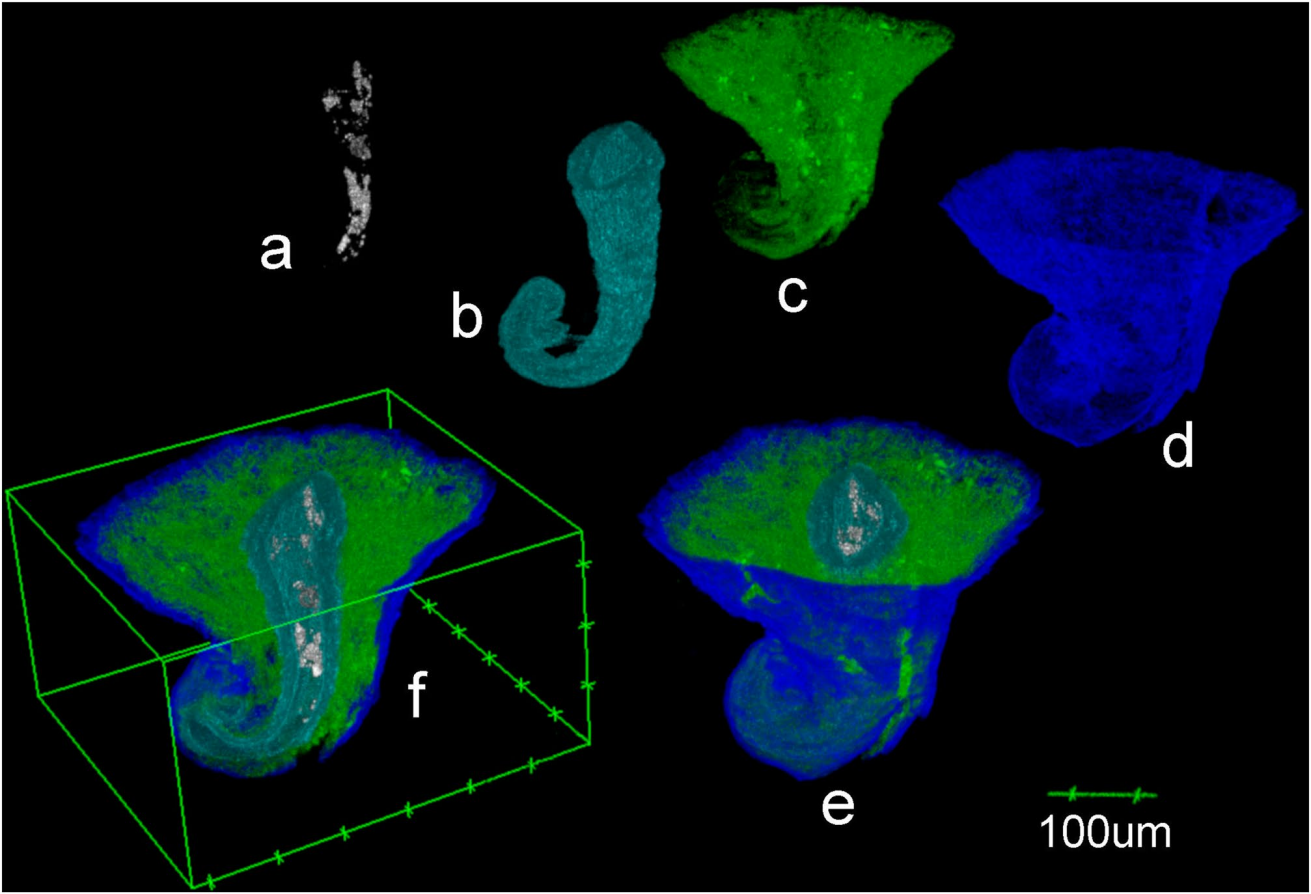
Volume reconstruction of (**a**) seed-embryo; (**b**) endocarp; (**c**) mesocarp; (**d**) exocarp; (**e**) complete composed volume; (**f**) longitudinally sectioned, from sample SMC_10 (CT Analyzer; ImageJ-FIJI, plug-in: 3D Viewer).

In X-ray MicroCT images, virtual longitudinal and transversal sections allow one to distinguish the three-layered pericarp in terms of the differences between the morphologies of their composing cells (Fig. 3b,c), very similar to those observed in HRLM images. In sample SMC_01, the limits between the layers are barely defined, resulting in a gradate transition from one cell morphology to another. Any relevant contrast level differences based on variations of the density values of layers are evident (Fig. 4c–f). This character makes the segmentation of images less evident, even when non-algorithmic procedures are used. A membranous tissue remnant of the seed-embryo structure is clearly observable inside the central cavity. It appears weakly attached to the inner side of the endocarp (Fig. 5) (Supplementary Video S1 online).

### Presence of cellular contents

Most of the exocarp cells show the presence of brown-reddish stained contents in the HRLM images. They could be associated with phenolic contents and/or pigments, commonly present in the skin of fruits (Fig. 4a). However, this characteristic is not observed in the X-ray MicroCT images, at least for the experimental parameters set for this study.

**Figure 6:**
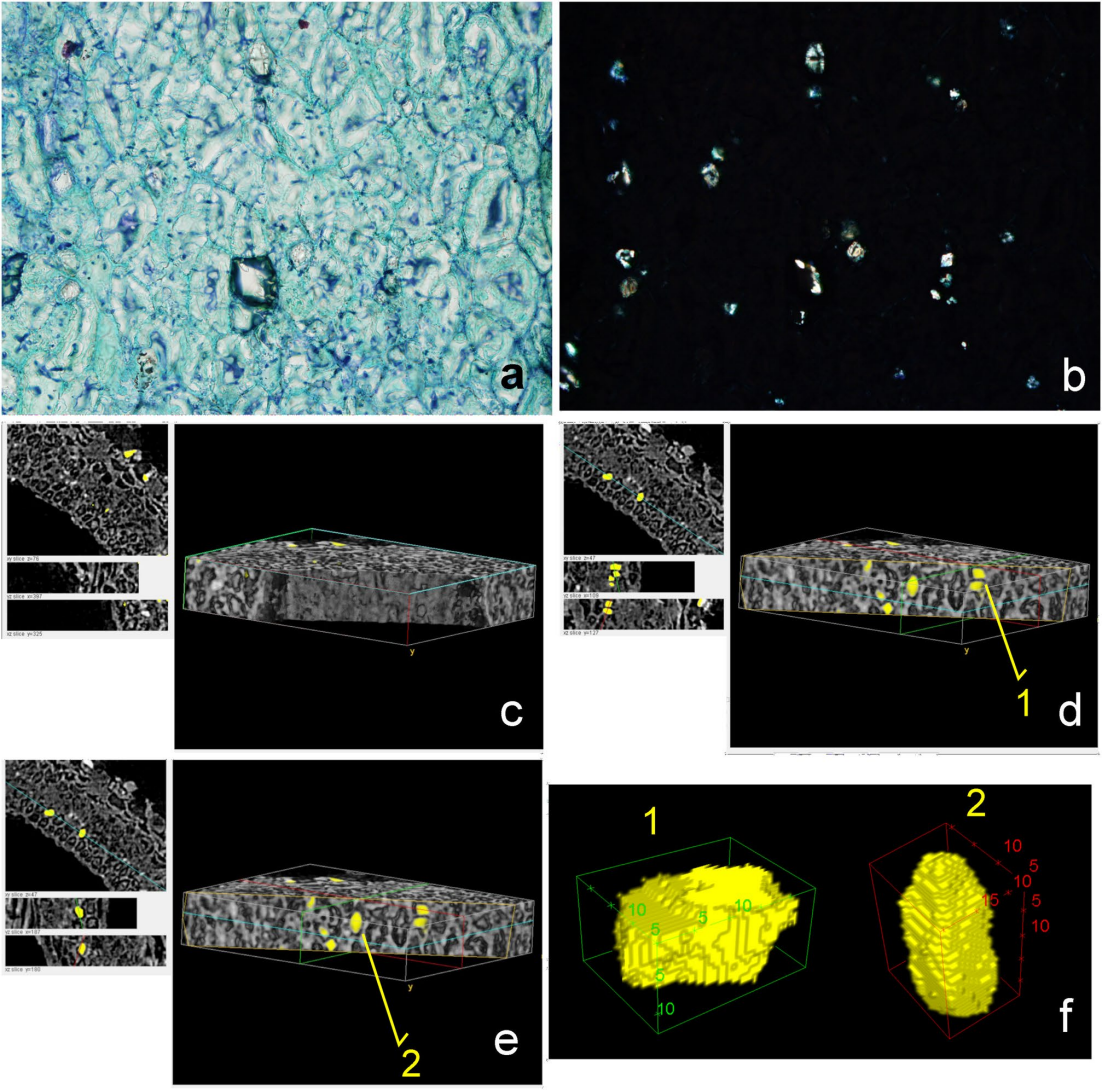
**(a**) Longitudinal section HRLM image from the inner layer region showing the thick walled cells, some of which contains crystals; (**b**) the same image evidencing the crystalline character of the particles under polarized light; (**c**) X-ray MicroCT reconstructed volume from a section of the endocarp of the sample SMC_01; the lines in blue, green and red indicate the position of the slices at the XY, XZ and YZ sections, respectively and their images appear on the left side; (**d**, **e**) the slices appear repositioned in order to reveal the localization of the particles inside the thick walled cells; the yellow lines in the volume indicate the clipping plane entering/cutting the volume; (**f**) reconstructed volumes from particles 1 and 2 (scale in μm) (CT Analyzer; ImageJ-FIJI, plug-ins: 3D Viewer and Volume Viewer).

**Figure 7:**
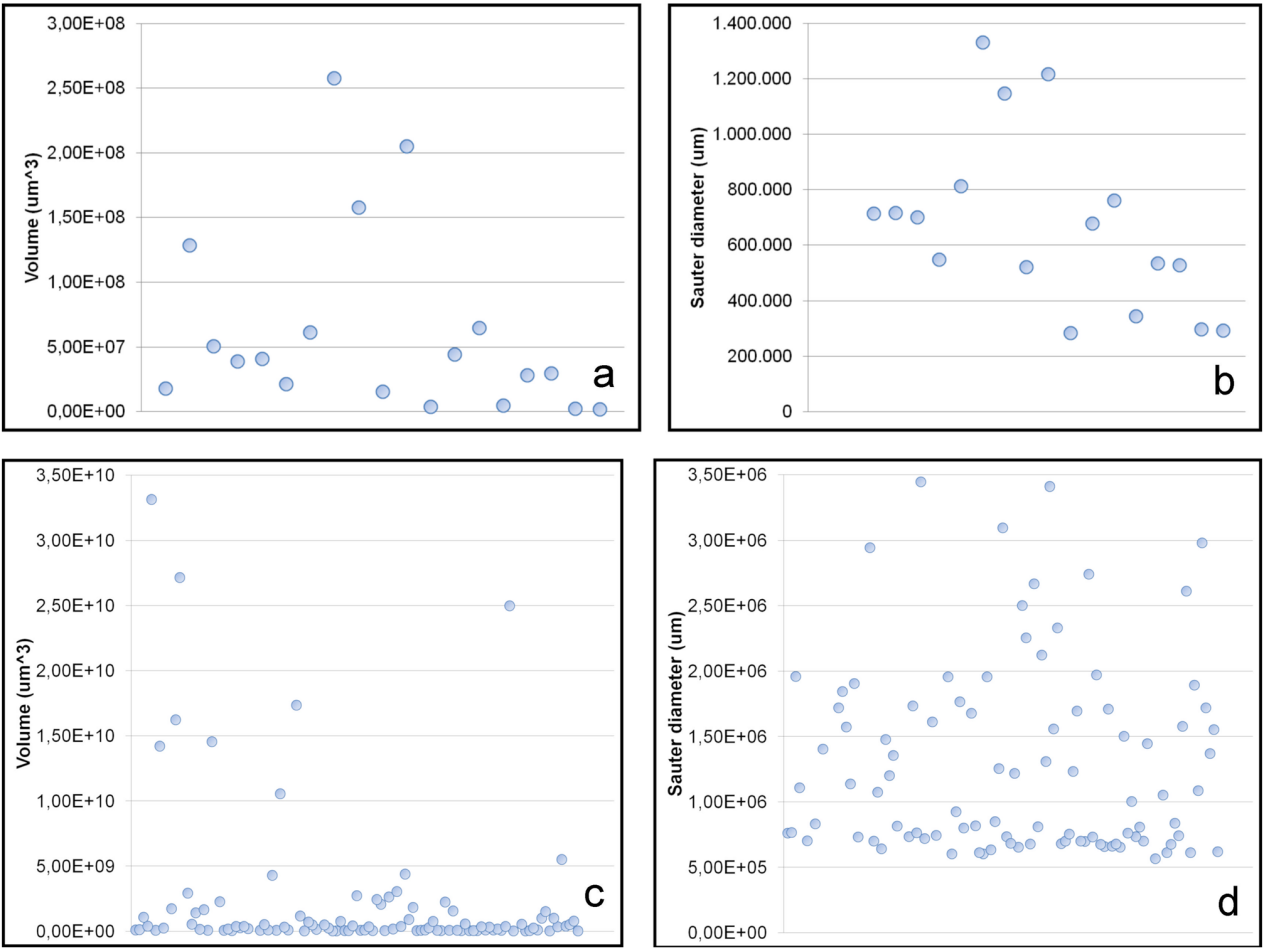
Volume and Sauter diameter distribution of particles in the endocarp (**a**, **b**) and mesocarp (**c**, **d**) of the sample SMC_01 (CT Analyzer).

HRLM also shows crystal contents in the cells of the endocarp. Some of these cells contain calcium oxalate crystals which exhibit the typical extinction cross under polarized light (Fig. 6a,b). These particles were also evidenced in X-ray MicroCT images, which exhibit several white and compacted particles in the inner layer of some cells. Their high X-ray attenuation coefficient makes them easily distinguishable from tissues and other particles in the samples because they are comparatively denser and their composition is very different from surrounding material (Fig. 6c–e). A total of 19 particles were segmented from a random section of the endocarp of sample SMC_01, most of them measuring between 1.50E+07 μm^3^ and 6.40 E+07 μm^3^ (Fig. 7a,b). The reconstructed volumes of some of them suggest crystal-like multi-faceted structures defined by edges and angles (Fig. 6f). Notwithstanding, the resolution of the X-ray microtomographies in this study is not high enough to confirm this morphology.

**Figure 8:**
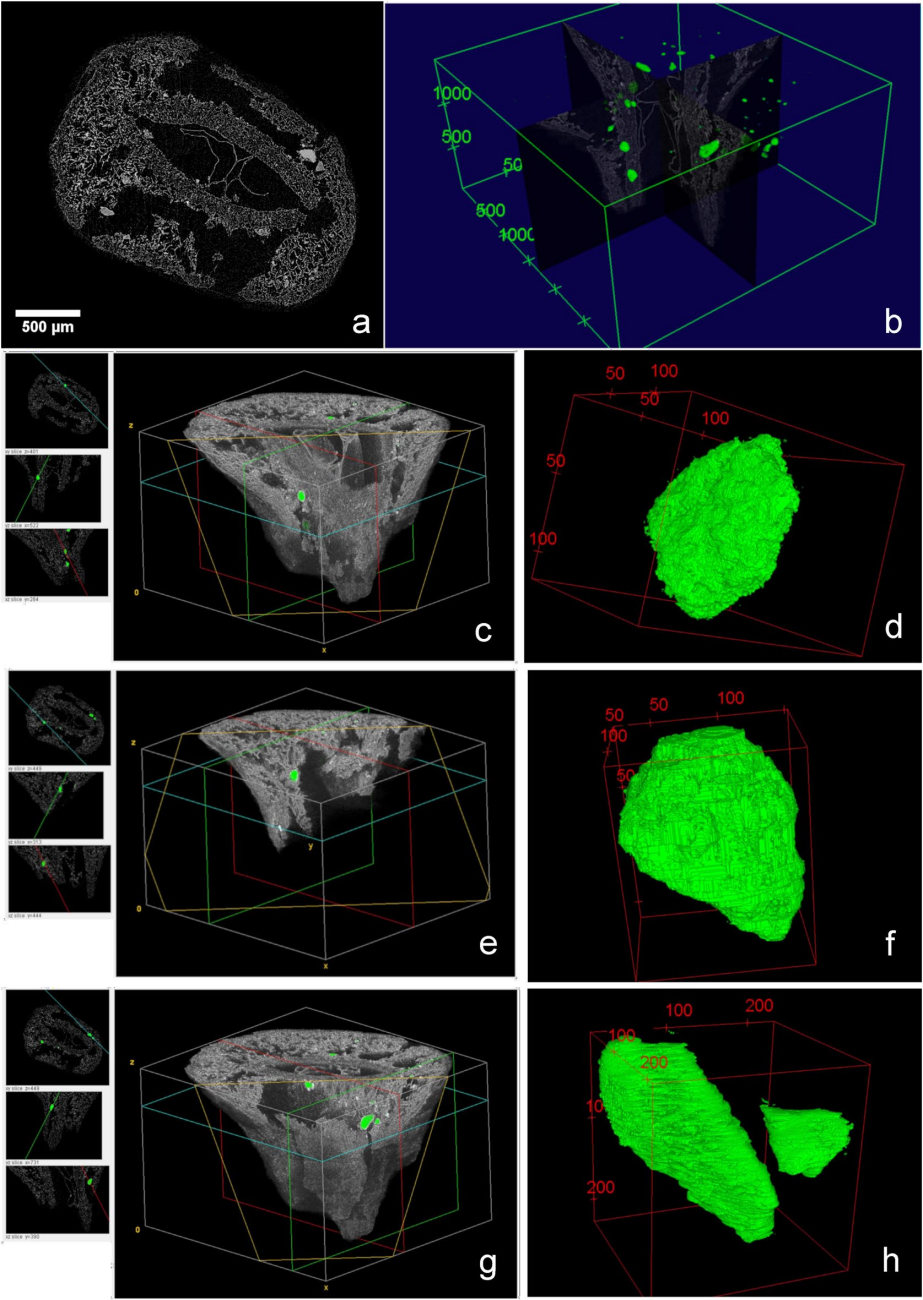
2D Visualization of dense amorphous particles in a virtual transversal section from sample SMC_01 (**a**) Volume reconstruction of amorphous particles in SMC_01 placed in the orthogonal section of the whole sample volume; (**b**) Volume reconstruction of the same sample showing the localization of three amorphous particles and their respective 3D image; (**c**–**e**) (CT Analyzer; ImageJ-FIJI, plug-ins: 3D Viewer and Volume Viewer).

X-ray MicroCT images also show a second type of dense particle present in the mesocarp and inside the cavity, next to the open pointy region of the samples. They do not seem to be directly associated with intracellular contents and their 3D morphological analysis shows a total of 111 particles which are bigger than the crystal ones. The volume of most of these particles is between 6.6 E+05 μm^3^ and 5.4 E+09 μm^3^, while others can reach 1.2 E+11 μm^3^ (Fig. 7c,d). Their Sauter diameter (the diameter of the sphere with the same volume/area ratio of a given particle) ranges between 5.6 E+05 μm^3^and 2.9 E+06 μm^3^. They moreover lack the multi-faceted morphology and might be better described as amorphous concretion-like structures (Fig. 8). The complete list of volume and Sauter diameter values, as well as other measured variables (‘Surface’, ‘Volume-equivalent sphere diameter’ and ‘Surface-equivalent sphere diameter’) can be accessed in the Supplementary Table S2 online.

### Vascularisation

**Figure 9:**
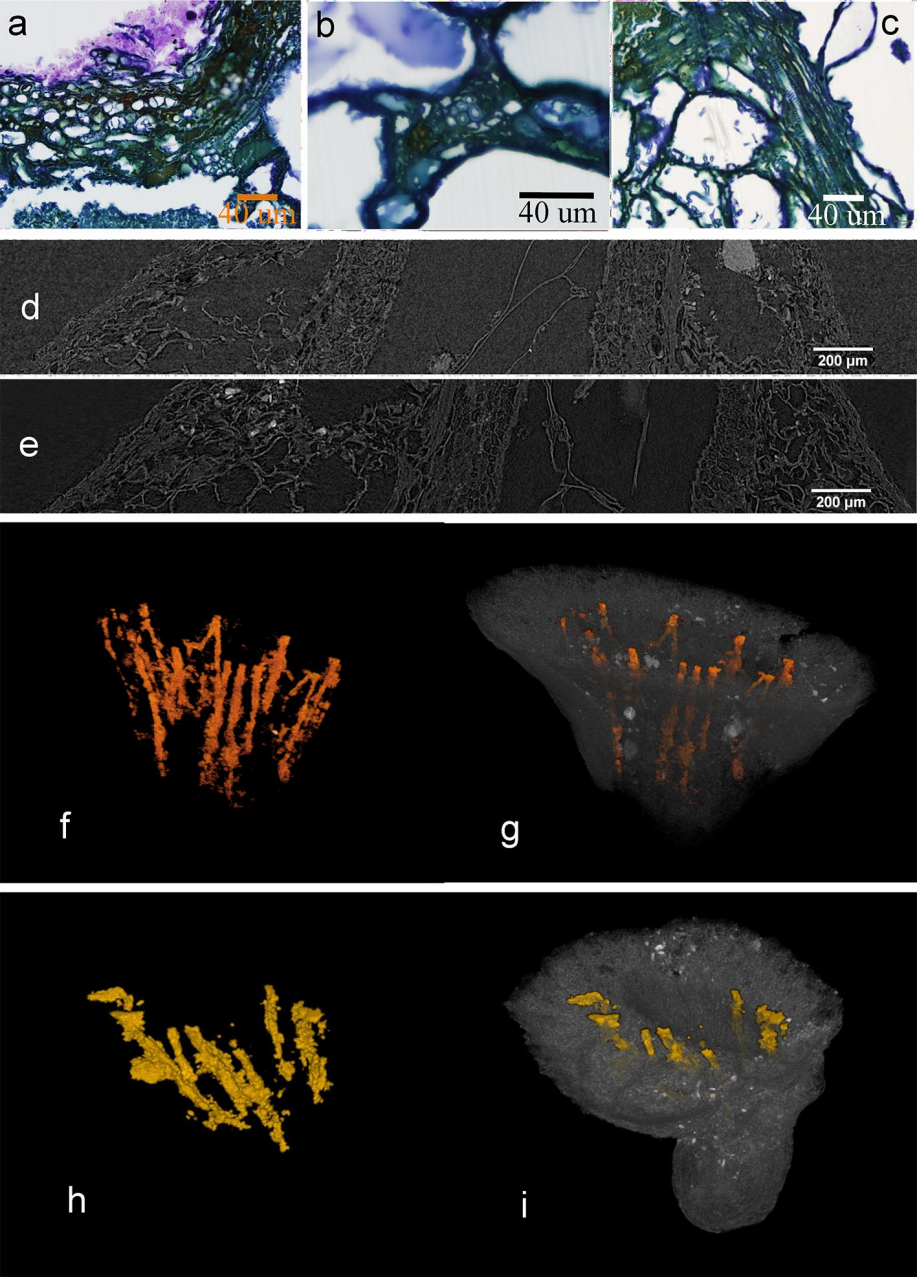
Vascular elements in microtomed longitudinal sections of SMC_11 (**a**–**c**); Vascular bundles as seen in X-ray MicroCT virtual longitudinal sections from samples SMC_01 (**d**) and SMC_10 (**e**); Volume reconstruction of a part of the vascularisation system in the region of the mesocarp-endocarp edge and their emplacement in exemplars SMC_01 (**f**, **g**) and SMC_10 (**h**, **i**) (CT Analyzer; ImageJ-FIJI, plug-ins: MorphoLibJ and 3D Viewer).

Several segments of the vascular system of the fruit can be observed in HRLM slices as vascular elements or fragments of bundles showing spiral thickened cell walls (Fig. 9a–c). They are present abundantly in the fleshy mesocarp layer, and are also morphologically distinguishable in X-ray MicroCT longitudinal virtual slices (Fig. 9d,e). The volumetric reconstruction of the more conspicuous bundles through the mesocarp (at least 12) reveals their distribution in the region near to the endocarp (Fig. 9f–i).

### Cavities and ducts

**Figure 10:**
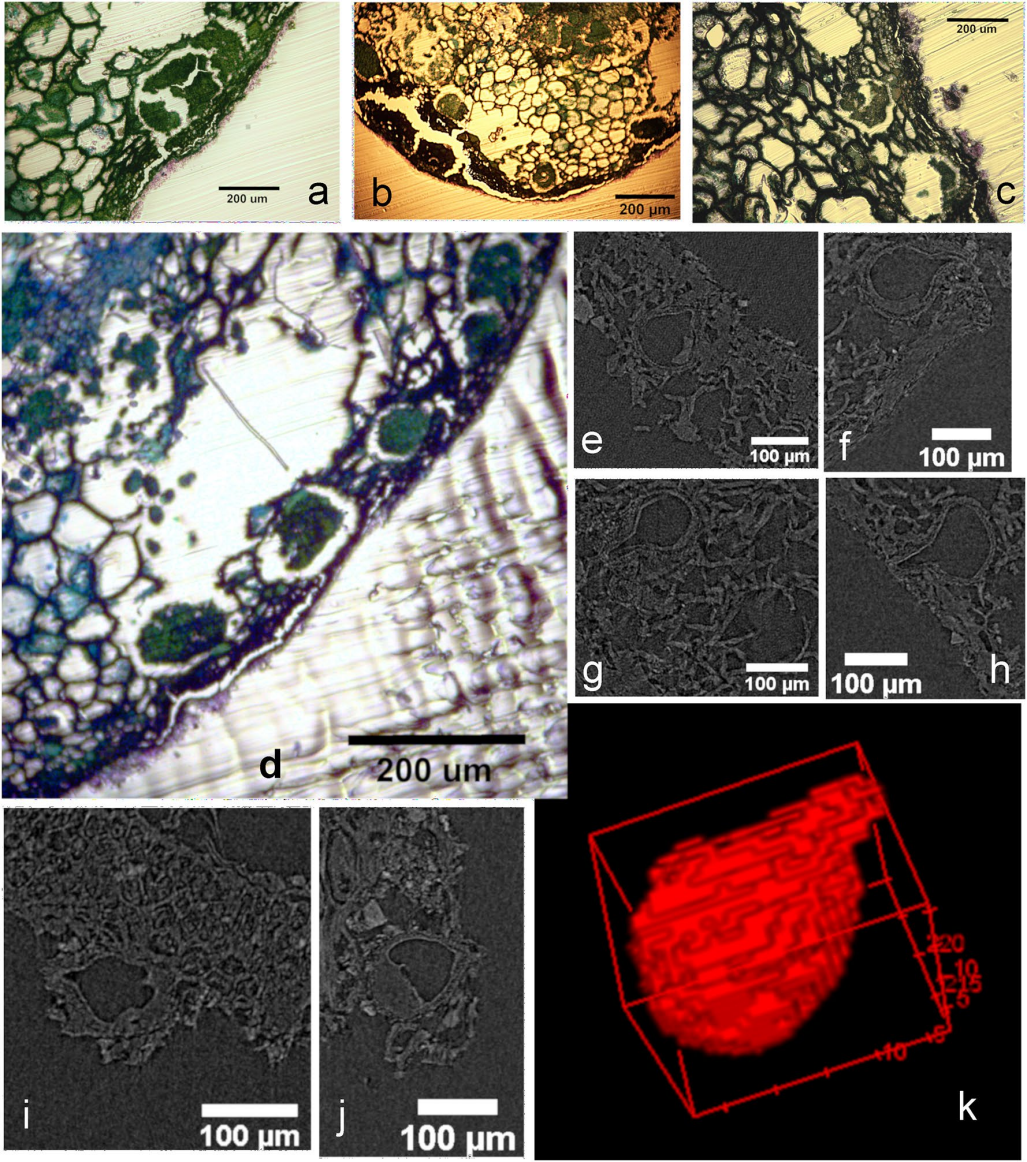
Sub-circular cavities observed in the microtomed longitudinal section from SMC_11 (**a**–**d**); Virtual X-ray MicroCT sections of cavities from sample SMC_01, suggesting their sub-circular/drop morphology (**e**–**j**); Volume reconstruction of one of these X-ray MicroCT imaged cavities (**k**) (CT Analyzer; ImageJ-FIJI, plug-in: 3D Viewer).

**Figure 11:**
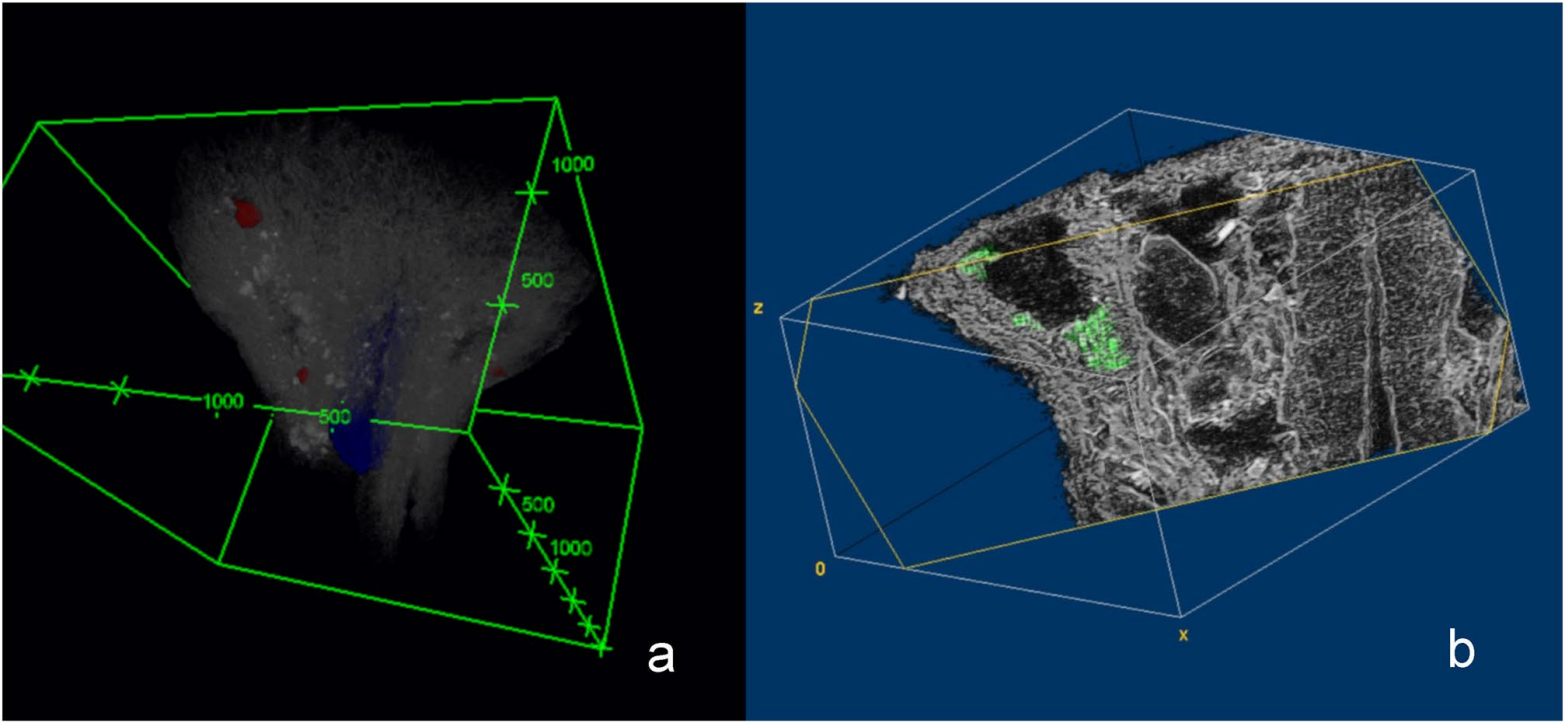
Volume reconstruction placing the cavities (in red) in the pericarp of sample SMC_01 (grey) with the endocarp highlighted in blue (**a**); Section on the volume reconstruction of the sample SMC_10 showing contents in a mesocarp secretory structure (**b**) (CTAnalyzer; ImageJ-FIJI, plug-ins: 3D Viewer and Volume Viewer).

Some microtomed longitudinal sections show several cavities with sub-circular contours which is compatible to those previously described for several species of the Anacardiaceae. The diameter of these structures ranges from 100 µm to 250 µm and some of them still obtrude with contents (Fig. 10a–d). They were observed less frequently in the distal half of the sample. Nonetheless, the microtomographied exemplars showed some of them in this position in both longitudinal and transversal virtual sections of the mesocarp (Fig. 10e–j). Contents are not evident in 2D virtual slices. Their size ranges from 80 µm to 100 µm in transversal and longitudinal diameter, according to their 3D sub-spherical/drop morphology (Figs. 10k and 11a).

Another type of structure with contents was also observed in the Micro-CT images, appearing as open spaces along the mesocarp, where some substances persist, probably due to their secretory function (Fig. 11b).

## Conclusions

The high resolution images obtained by both techniques were able to evidence each of the studied morphological traits in the archaeobotanical samples from Monte Castelo. HRLM and X-ray MicroCT are able to recognize the three differentiated layers of tissues composing the pericarp of the fruit and some of its most relevant histological characteristics: the presence of vascular tissues in the mesocarp; the existence of mineral particles within and between the tissues; and the occurrence of secretory structures. However, it is possible to point out some differences between the information obtained through each method.

The clear contours and detailed definition of the objects in the HRLM images are among their most notable characteristics. The contrast between the different colors and color shades induced by staining reinforces these effects. HRLM allowed the visualization of very fine structures in vascular tissues, cell walls and contents, which were either barely or not visible in the X-ray MicroCT images.

Similarly, the use of polarized light coupled to HRLM enhances the analysis of mineral particles, giving a clear idea about their crystalline nature. This was not possible using microtomography. Despite this, the reconstructed volumes of the particles show differences between faceted and non-faceted morphologies, suggesting that a morphological analysis of crystalline minerals could be achieved focusing on appropriate X-ray MicroCT experimental settings.

Indeed, the possibility of performing 3D reconstructions of samples and visualizing their internal structure in a three-dimensional space allows the user to reveal the position and distribution of vascular bundles, mineral particles and secretory structures. Moreover, dynamic and non-destructive virtual slicing addresses the full range of slicing planes describing characteristics in a way that is not available to HRLM static slicing.

The non-destructive character of X-ray MicroCT helped to describe the presence of remnant tissues inside the central cavity of the samples which are absent in the microscopy images and probably represent the seed-embryo structures. As shown by microtomography, these structures are weakly tied to the rest of the fruit and could be detached and lost from the microtomed-for-microscopy sample. Likewise, some differences in the average size of the secretory structures were observed when measured by X-ray MicroCT or HRLM images. This effect could be linked to distortions originating in sample preparation procedures, including sample dehydration and resin embedding, meaning all quantitative comparisons between specimens should be made under the same imaging method^53^.

Results obtained from both imaging methods are complementary in terms of a more exhaustive analysis of morphological characteristics of plant remains. However, X-ray MicroCT represents a suitable alternative analytic technique when sample preservation is required, for example when it is a rare, fragile specimen, or it will later be intended for further analysis. Based on the results of this study, the application of this analytical technique presents two types of challenge.

The first is of a technical nature, and has to do with the limitations of the technique in defining contours and subtle definition of details when applied to biological materials. Beyond the loss of information that this may represent for the study of plant remains, its resolution depends fundamentally on the technological development of X-ray MicroCT techniques, new instruments and even more advanced X-ray microscopy modalities such as ptychography.

The second challenge is a methodological one, linked to some basic procedures of archaeobotanical studies. A good part of Archaeobotany research is focused on taxonomic identification of archaeological plant remains. This task is based primarily on morphological comparative analysis between old specimens and contemporary plant reference material. These references may be previous bibliographical studies, modern described plant exemplars itself or, as is generally the case, a combination of both. However, the analytical parameters used by morphology and plant anatomy to describe different plant taxa have been constructed based on two-dimensional, light or SEM microscopy images.

This means that a large number of the variables used to describe and compare plant material, relies on images of a different nature than three-dimensional X-ray MicroCT. It is therefore necessary to consider providing three-dimensional descriptions of reference plant material to archaeobotanical studies using this technique. Nonetheless, it is worth mentioning that virtual slicing would offer two-dimensional images equivalent to Light Microscopy, but properly related to volume, which could pave the way for new taxonomical standards.

The present study has shed light on some differences observed between analytic images based on the variations of the light reflected by the object (HRLM) from those based on the variations of the X-ray attenuation coefficient (and phase shifts) of the irradiated object (X-ray phase-contrast MicroCT). A more detailed analysis of these differences, their impact on the recognition and comparability of specific morphological characteristics, and the production of three-dimensional reference material, will be valuable in assisting the implementation of X-ray MicroCT to the study of archaeological plant remains.

This research benefitted from resources at the Brazilian Synchrotron Light Laboratory (LNLS), an open national facility operated by the Brazilian Centre for Research in Energy and Materials (CNPEM) for the Brazilian Ministry for Science, Technology, Innovations and Communications (MCTIC). The IMX Beamline staff is acknowledged for the assistance during the experiments. We are also thankful to the Laboratory of Complex Fluids of the Institute of Physics (IF), University of Sao Paulo (USP), for microscopy sessions, especially to Dennys Reis for technical support with microscopy images.

## Supplementary information

Supplementary Video

Supplementary Table
