# Supplementary figures and images for: A correlation analysis of Light Microscopy and X-ray MicroCT imaging methods applied to archaeological plant remains’ morphological attributes visualization
Source: Sci Rep. 2020 Sep 15;10:15105. doi: 10.1038/s41598-020-71726-z (PMC7493802; doi:10.1038/s41598-020-71726-z)

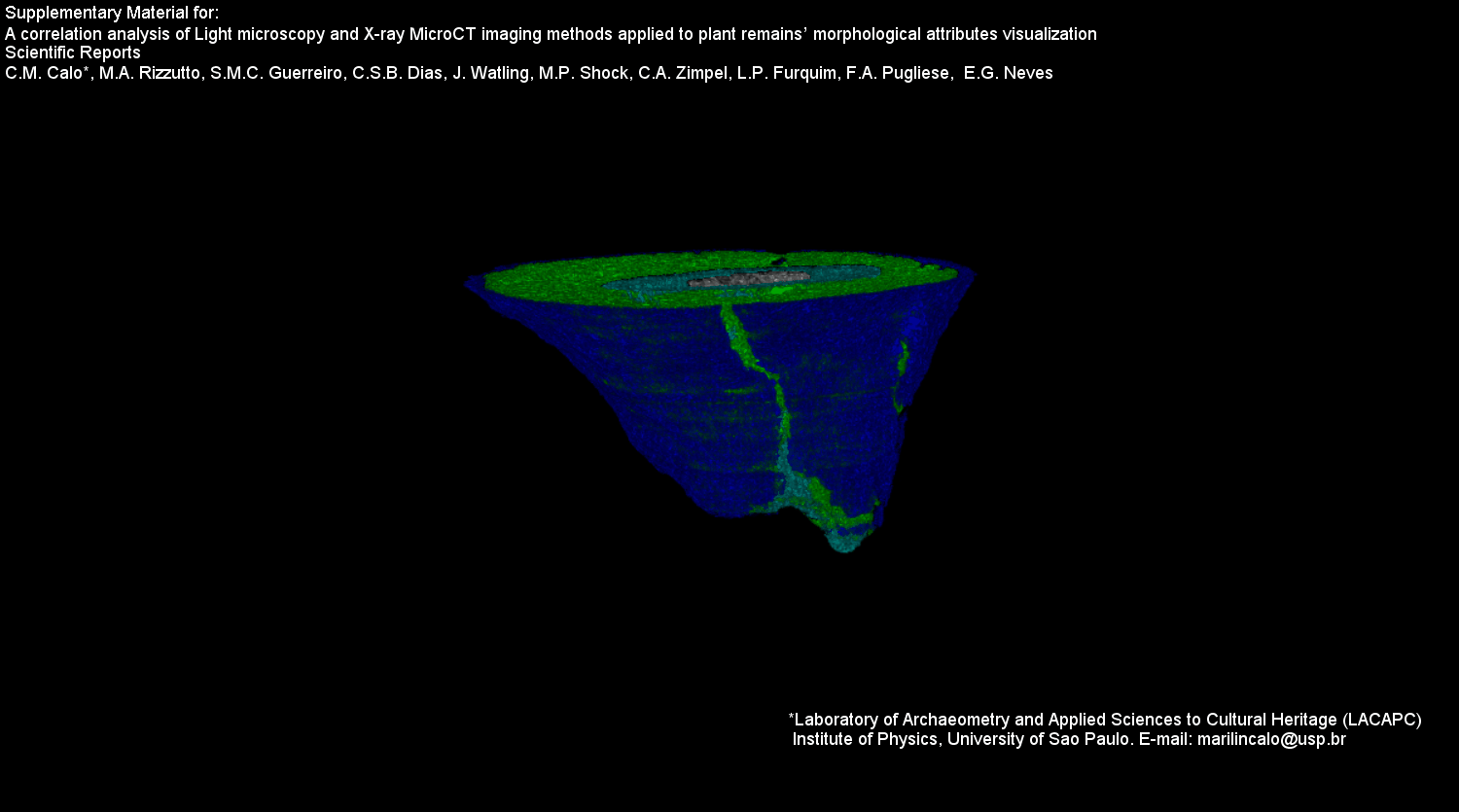

Supplement: Supplementary file 1 — Supplementary Video [file 41598_2020_71726_MOESM1_ESM.gif]
