# Supplementary material for: A correlation analysis of Light Microscopy and X-ray MicroCT imaging methods applied to archaeological plant remains’ morphological attributes visualization
Source: Sci Rep. 2020 Sep 15;10:15105. doi: 10.1038/s41598-020-71726-z (PMC7493802; doi:10.1038/s41598-020-71726-z)
Supplement: Supplementary file 2 — Supplementary Table [file 41598_2020_71726_MOESM2_ESM.docx]

**Supplementary Material for:**

A correlation analysis of Light microscopy and X-ray MicroCT imaging methods applied to plant remains’ morphological attributes visualization

Journal of Archaeological Methods and Theory

C.M. Calo*, M.A. Rizzutto, S.M.C. Guerreiro, C.S.B. Dias, J. Watling, M.P. Shock, C.A. Zimpel, L.P. Furquim, F.A. Pugliese, E.G. Neves

*Laboratory of Archaeometry and Applied Sciences to Cultural Heritage (LACAPC), Institute of Physics, University of Sao Paulo. E-mail: [marilincalo@usp.br](mailto:marilincalo@usp.br)

| **Mesocarp particles measures** | | | | | | |
| --- | --- | --- | --- | --- | --- | --- |
| **#** | **Object volume**  **(uµ^3^)** | **Object surface**  **(uµ^2^)** | **Volume-equivalent sphere diameter**  **(µ)** | **Surface-equivalent sphere diameter**  **(uµ)** | **Sauter diameter**  **(uµ)** | **Sphericity** |
| 1 | 97.072.282 | 76.338.245 | 1.228.473 | 1.558.821 | 762.964 | 0.62107 |
| 2 | 107.011.423 | 83.551.916 | 1.269.045 | 1.630.810 | 768.467 | 0.60555 |
| 3 | 1.056.033.784 | 323.158.638 | 2.722.031 | 3.207.251 | 1.960.710 | 0.72031 |
| 4 | 383.319.557 | 207.482.028 | 1.941.724 | 2.569.895 | 1.108.490 | 0.57088 |
| 5 | 33.156.942.911 | 3.382.704.435 | 8.587.339 | 10.376.648 | 5.881.142 | 0.68486 |
| 6 | 57.315.716 | 48.781.395 | 1.030.604 | 1.246.098 | 704.970 | 0.68404 |
| 7 | 14.199.355.720 | 4.677.975.592 | 6.472.767 | 12.202.647 | 1.821.218 | 0.28137 |
| 8 | 226.927.166 | 163.562.154 | 1.630.407 | 2.281.742 | 832.444 | 0.51057 |
| 9 | 52.182.000.308 | 5.945.305.277 | 9.988.663 | 13.756.633 | 5.266.206 | 0.52722 |
| 10 | 1.734.396.757 | 740.163.345 | 3.211.564 | 4.853.878 | 1.405.957 | 0.43778 |
| 11 | 16.225.168.106 | 2.223.751.772 | 6.767.011 | 8.413.336 | 4.377.782 | 0.64693 |
| 12 | 27.144.805.914 | 3.170.752.147 | 8.033.338 | 10.046.302 | 5.136.599 | 0.63941 |
| 13 | 662.609 | 2.017.591 | 233.034 | 253.420 | 197.050 | 0.84558 |
| 14 | 2.930.042.349 | 1.021.777.006 | 3.824.944 | 5.702.997 | 1.720.557 | 0.44983 |
| 15 | 542.842.778 | 176.515.796 | 2.180.512 | 2.370.374 | 1.845.193 | 0.84622 |
| 16 | 1.412.318.877 | 537.943.598 | 2.999.011 | 4.138.028 | 1.575.242 | 0.52525 |
| 17 | 141.964.071 | 74.804.709 | 1.394.419 | 1.543.084 | 1.138.678 | 0.81660 |
| 18 | 1.662.487.068 | 523.285.843 | 3.166.551 | 4.081.263 | 1.906.209 | 0.60198 |
| 19 | 68.248.772 | 55.882.801 | 1.092.359 | 1.333.718 | 732.770 | 0.67081 |
| 20 | 14.540.798.361 | 2.053.155.011 | 6.524.239 | 8.084.179 | 4.249.304 | 0.65131 |
| 21 | 39.170.819.258 | 3.250.715.395 | 9.077.955 | 10.172.192 | 7.229.944 | 0.79643 |
| 22 | 2.270.265.571 | 462.602.576 | 3.513.118 | 3.837.329 | 2.944.556 | 0.83816 |
| 23 | 56.321.802 | 48.153.190 | 1.024.612 | 1.238.048 | 701.783 | 0.68493 |
| 24 | 163.167.573 | 90.985.276 | 1.460.646 | 1.701.808 | 1.076.004 | 0.73666 |
| 25 | 35.449.605 | 33.097.644 | 878.087 | 1.026.416 | 642.637 | 0.73186 |
| 26 | 354.496.047 | 143.730.981 | 1.891.781 | 2.138.948 | 1.479.831 | 0.78224 |
| 27 | 255.104.632 | 127.361.761 | 1.695.274 | 2.013.467 | 1.201.795 | 0.70891 |
| 28 | 368.907.802 | 163.135.050 | 1.917.078 | 2.278.761 | 1.356.819 | 0.70775 |
| 29 | 182.548.899 | 134.297.599 | 1.516.329 | 2.067.565 | 815.572 | 0.53786 |
| 30 | 74.846.705.060 | 6.091.392.368 | 11.264.831 | 13.924.620 | 7.372.374 | 0.65446 |
| 31 | 120.166.490.163 | 8.878.857.365 | 13.190.510 | 16.811.389 | 8.120.402 | 0.61562 |
| 32 | 72.224.428 | 58.952.985 | 1.113.171 | 1.369.866 | 735.071 | 0.66034 |
| 33 | 504.080.126 | 174.350.801 | 2.127.324 | 2.355.793 | 1.734.710 | 0.81544 |
| 34 | 98.066.196 | 76.966.450 | 1.232.651 | 1.565.221 | 764.485 | 0.62020 |
| 35 | 4.273.333.888 | 743.854.741 | 4.337.666 | 4.865.967 | 3.446.910 | 0.79465 |
| 36 | 64.273.115 | 53.553.087 | 1.070.722 | 1.305.622 | 720.105 | 0.67254 |
| 37 | 10.554.043.053 | 1.369.612.762 | 5.863.273 | 6.602.736 | 4.623.515 | 0.78856 |
| 38 | 317.058.614 | 117.875.640 | 1.822.693 | 1.937.033 | 1.613.863 | 0.88543 |
| 39 | 86.139.226 | 69.236.839 | 1.180.504 | 1.484.546 | 746.475 | 0.63234 |
| 40 | 49.169.082.084 | 4.628.302.666 | 9.792.596 | 12.137.687 | 6.374.140 | 0.65091 |
| 41 | 17.338.517.606 | 2.352.036.096 | 6.918.381 | 8.652.609 | 4.423.023 | 0.63931 |
| 42 | 1.156.750.418 | 354.545.717 | 2.805.953 | 3.359.396 | 1.957.576 | 0.69765 |
| 43 | 28.492.206 | 28.325.951 | 816.412 | 949.549 | 603.522 | 0.73924 |
| 44 | 699.715.562 | 453.098.381 | 2.373.054 | 3.797.706 | 926.574 | 0.39046 |
| 45 | 489.171.414 | 166.113.169 | 2.106.141 | 2.299.467 | 1.766.885 | 0.83892 |
| 46 | 145.774.075 | 109.182.728 | 1.406.783 | 1.864.241 | 801.083 | 0.56944 |
| 47 | 8.613.923 | 11.677.201 | 547.941 | 609.669 | 442.602 | 0.80775 |
| 48 | 477.078.792 | 170.408.389 | 2.088.641 | 2.329.006 | 1.679.772 | 0.80424 |
| 49 | 234.232.435 | 171.622.363 | 1.647.718 | 2.337.287 | 818.888 | 0.49698 |
| 50 | 31.473.948 | 30.767.930 | 843.952 | 989.633 | 613.768 | 0.72725 |
| 51 | 28.492.206 | 28.325.951 | 816.412 | 949.549 | 603.522 | 0.73924 |
| 52 | 744.773.003 | 228.396.713 | 2.422.935 | 2.696.311 | 1.956.525 | 0.80750 |
| 53 | 32.467.862 | 30.655.665 | 852.743 | 987.826 | 635.469 | 0.74521 |
| 54 | 54.002.669 | 38.097.825 | 1.010.351 | 1.101.223 | 850.484 | 0.84177 |
| 55 | 412.308.720 | 196.804.823 | 1.989.488 | 2.502.897 | 1.257.008 | 0.63182 |
| 56 | 2.717.029.981 | 526.632.161 | 3.729.912 | 4.094.291 | 3.095.553 | 0.82993 |
| 57 | 75.206.171 | 61.394.963 | 1.128.284 | 1.397.949 | 734.974 | 0.65141 |
| 58 | 89.617.926 | 78.696.591 | 1.196.186 | 1.582.716 | 683.267 | 0.57120 |
| 59 | 329.648.193 | 162.114.344 | 1.846.506 | 2.271.621 | 1.220.058 | 0.66074 |
| 60 | 41.413.090 | 37.981.600 | 924.797 | 1.099.542 | 654.208 | 0.70741 |
| 61 | 2.441.218.805 | 585.449.765 | 3.599.173 | 4.316.879 | 2.501.891 | 0.69513 |
| 62 | 2.064.525.342 | 548.860.099 | 3.403.616 | 4.179.804 | 2.256.887 | 0.66309 |
| 63 | 46.382.660 | 40.939.519 | 960.400 | 1.141.554 | 679.773 | 0.70780 |
| 64 | 2.622.939.442 | 589.847.059 | 3.686.350 | 4.333.061 | 2.668.088 | 0.72377 |
| 65 | 173.603.672 | 128.269.497 | 1.491.146 | 2.020.630 | 812.057 | 0.54459 |
| 66 | 3.038.809.687 | 858.093.557 | 3.871.699 | 5.226.277 | 2.124.810 | 0.54881 |
| 67 | 348.863.867 | 159.849.814 | 1.881.709 | 2.255.699 | 1.309.469 | 0.69589 |
| 68 | 4.392.437.933 | 772.446.726 | 4.377.596 | 4.958.603 | 3.411.838 | 0.77939 |
| 69 | 908.768.838 | 349.419.947 | 2.589.117 | 3.335.024 | 1.560.476 | 0.60271 |
| 70 | 1.830.624.212 | 471.282.194 | 3.269.893 | 3.873.161 | 2.330.609 | 0.71275 |
| 71 | 49.364.403 | 43.381.498 | 980.555 | 1.175.107 | 682.748 | 0.69629 |
| 72 | 56.321.802 | 48.153.190 | 1.024.612 | 1.238.048 | 701.783 | 0.68493 |
| 73 | 97.072.282 | 77.078.715 | 1.228.473 | 1.566.363 | 755.635 | 0.61510 |
| 74 | 266.037.687 | 129.233.647 | 1.719.154 | 2.028.210 | 1.235.147 | 0.71846 |
| 75 | 746.429.527 | 263.846.480 | 2.424.730 | 2.898.016 | 1.697.418 | 0.70004 |
| 76 | 56.321.802 | 48.153.190 | 1.024.612 | 1.238.048 | 701.783 | 0.68493 |
| 77 | 53.340.059 | 45.711.212 | 1.006.201 | 1.206.248 | 700.135 | 0.69582 |
| 78 | 2.233.490.747 | 488.511.220 | 3.494.045 | 3.943.323 | 2.743.221 | 0.78511 |
| 79 | 71.230.514 | 58.324.780 | 1.108.041 | 1.362.547 | 732.764 | 0.66132 |
| 80 | 1.549.180.855 | 470.970.041 | 3.092.914 | 3.871.878 | 1.973.604 | 0.63811 |
| 81 | 73.549.647 | 65.230.288 | 1.119.938 | 1.440.953 | 676.523 | 0.60407 |
| 82 | 42.407.004 | 38.609.806 | 932.137 | 1.108.597 | 659.009 | 0.70699 |
| 83 | 565.040.194 | 198.271.334 | 2.209.837 | 2.512.205 | 1.709.900 | 0.77377 |
| 84 | 45.388.746 | 41.051.784 | 953.491 | 1.143.118 | 663.388 | 0.69575 |
| 85 | 46.382.660 | 40.939.519 | 960.400 | 1.141.554 | 679.773 | 0.70780 |
| 86 | 39.425.261 | 36.167.827 | 909.757 | 1.072.967 | 654.039 | 0.71892 |
| 87 | 341.078.206 | 136.112.079 | 1.867.605 | 2.081.486 | 1.503.518 | 0.80505 |
| 88 | 104.029.681 | 81.850.407 | 1.257.147 | 1.614.119 | 762.584 | 0.60660 |
| 89 | 319.543.399 | 190.979.351 | 1.827.443 | 2.465.575 | 1.003.910 | 0.54935 |
| 90 | 85.145.312 | 69.349.104 | 1.175.946 | 1.485.749 | 736.667 | 0.62645 |
| 91 | 174.597.586 | 129.638.172 | 1.493.986 | 2.031.382 | 808.084 | 0.54089 |
| 92 | 56.321.802 | 48.153.190 | 1.024.612 | 1.238.048 | 701.783 | 0.68493 |
| 93 | 353.005.176 | 146.399.894 | 1.889.126 | 2.158.716 | 1.446.744 | 0.76583 |
| 94 | 24.981.866.506 | 3.130.038.152 | 7.814.036 | 9.981.593 | 4.788.798 | 0.61285 |
| 95 | 18.553.064 | 19.631.341 | 707.629 | 790.497 | 567.044 | 0.80133 |
| 96 | 662.609 | 2.017.591 | 233.034 | 253.420 | 197.050 | 0.84558 |
| 97 | 539.529.731 | 307.522.305 | 2.176.067 | 3.128.696 | 1.052.665 | 0.48375 |
| 98 | 31.473.948 | 30.767.930 | 843.952 | 989.633 | 613.768 | 0.72725 |
| 99 | 43.400.918 | 38.497.541 | 939.363 | 1.106.985 | 676.421 | 0.72008 |
| 100 | 242.183.748 | 173.503.018 | 1.666.155 | 2.350.058 | 837.508 | 0.50266 |
| 101 | 88.127.055 | 71.050.612 | 1.189.516 | 1.503.865 | 744.205 | 0.62564 |
| 102 | 1.000.540.244 | 380.307.724 | 2.673.491 | 3.479.306 | 1.578.522 | 0.59043 |
| 103 | 1.515.719.079 | 348.197.203 | 3.070.483 | 3.329.183 | 2.611.829 | 0.85062 |
| 104 | 31.473.948 | 30.767.930 | 843.952 | 989.633 | 613.768 | 0.72725 |
| 105 | 992.257.626 | 314.429.594 | 2.666.093 | 3.163.638 | 1.893.443 | 0.71019 |
| 106 | 325.506.884 | 179.505.969 | 1.838.741 | 2.390.367 | 1.088.009 | 0.59171 |
| 107 | 5.495.848.292 | 1.105.810.853 | 4.717.139 | 5.932.879 | 2.981.983 | 0.63216 |
| 108 | 390.442.609 | 136.112.079 | 1.953.677 | 2.081.486 | 1.721.123 | 0.88097 |
| 109 | 495.963.161 | 217.082.676 | 2.115.844 | 2.628.680 | 1.370.804 | 0.64788 |
| 110 | 769.952.161 | 296.969.644 | 2.449.938 | 3.074.547 | 1.555.618 | 0.63496 |
| 111 | 28.492.206 | 27.585.482 | 816.412 | 937.056 | 619.722 | 0.75908 |

| **Endocarp particles measures** | | | | | | |
| --- | --- | --- | --- | --- | --- | --- |
| **#** | **Object volume**  **(uµ^3^)** | **Object surface**  **(uµ^2^)** | **Volume-equivalent sphere diameter**  **(µ)** | **Surface-equivalent sphere diameter**  **(uµ)** | **Sauter diameter**  **(uµ)** | **Sphericity** |
| 1 | 17.708.102 | 19.874.231 | 696.719 | 795.372 | 534.605 | 0.76732 |
| 2 | 128.475.636 | 71.639.605 | 1.348.779 | 1.510.086 | 1.076.016 | 0.79777 |
| 3 | 50.383.089 | 42.368.207 | 987.254 | 1.161.302 | 713.503 | 0.72272 |
| 4 | 39.011.583 | 32.716.274 | 906.564 | 1.020.486 | 715.453 | 0.78919 |
| 5 | 40.840.287 | 35.007.112 | 920.513 | 1.055.609 | 699.977 | 0.76042 |
| 6 | 21.257.534 | 23.262.634 | 740.465 | 860.507 | 548.284 | 0.74046 |
| 7 | 61.070.898 | 45.096.882 | 1.052.637 | 1.198.114 | 812.529 | 0.77190 |
| 8 | 257.757.188 | 116.153.778 | 1.701.130 | 1.922.834 | 1.331.462 | 0.78269 |
| 9 | 157.615.205 | 82.491.305 | 1.443.887 | 1.620.426 | 1.146.413 | 0.79398 |
| 10 | 15.355.599 | 17.639.793 | 664.389 | 749.328 | 522.305 | 0.78614 |
| 11 | 205.021.596 | 101.121.433 | 1.576.160 | 1.794.100 | 1.216.487 | 0.77180 |
| 12 | 3.981.336 | 8.391.009 | 423.653 | 516.811 | 284.686 | 0.67198 |
| 13 | 44.060.966 | 38.906.766 | 944.101 | 1.112.853 | 679.485 | 0.71972 |
| 14 | 64.627.222 | 50.955.313 | 1.072.685 | 1.273.561 | 760.987 | 0.70942 |
| 15 | 4.555.678 | 7.917.689 | 443.117 | 502.024 | 345.228 | 0.77909 |
| 16 | 27.935.289 | 31.370.421 | 811.057 | 999.275 | 534.299 | 0.65877 |
| 17 | 29.636.030 | 33.665.215 | 827.194 | 1.035.180 | 528.190 | 0.63853 |
| 18 | 2.216.959 | 4.462.100 | 348.540 | 376.873 | 298.105 | 0.85530 |
| 19 | 2.069.927 | 4.255.553 | 340.658 | 368.047 | 291.844 | 0.85671 |
